# Editorial: Fighting antimicrobial resistance in developing countries: innovative approaches and challenges

**DOI:** 10.3389/fcimb.2024.1542233

**Published:** 2025-01-09

**Authors:** Tugce Katipoglu-Yazan, Ilke Pala-Ozkok, Si-Kyung Cho, Cansu Uluseker

**Affiliations:** ^1^ Environmental Engineering Department, Faculty of Civil Engineering, Istanbul Technical University, Istanbul, Türkiye; ^2^ Department of Chemistry, Bioscience and Environmental Engineering, Faculty of Science and Technology, University of Stavanger, Stavanger, Norway; ^3^ Department of Biological and Environmental Science, Dongguk University, Seoul, Republic of Korea; ^4^ UK Centre for Ecology & Hydrology, Lancaster, United Kingdom

**Keywords:** antimicrobial resistance (AMR), developing countries, multidrug resistance, environmental contamination, one health

Antimicrobial resistance (AMR) has emerged as a global health concern, particularly influencing developing countries. Limited healthcare infrastructure, socio-economic disparities, and cultural practices complicates efforts to combat AMR in these regions. *Fighting Antimicrobial Resistance in Developing Countries: Innovative Approaches and Challenges* features eight Research Topics giving critical insights into the molecular epidemiology, environmental factors, and public health issues of AMR and highlights the urgent need for integrated, multidisciplinary strategies.

In developing countries, the necessity of robust surveillance systems to monitor AMR trends and resistance mechanisms were highlighted by several contributors. Wei et al. provided a detailed analysis of *Helicobacter pylori* resistance patterns in Nanjing, China and revealed significant multidrug resistance rates, especially against metronidazole and levofloxacin. As a conclusion, they suggested implementation of genotypic resistance testing to enhance related treatment efficacy. Similarly, Li et al. presented molecular epidemiological data on *Klebsiella pneumoniae* in China, identifying carbapenem-resistant strains harboring blaKPC-2 and blaNDM genes. These studies emphasized the critical role of molecular surveillance in developing targeted and effective strategies against AMR across clinical and public health domains.

The transmission of AMR through animals and the environment is another theme explored in this Research Topic. Tartor et al. reported the presence of pan drug-resistant carbapenemase-producing *Enterobacterales* in dogs and cats in Egypt. In their work, the potential for cross-species transmission of AMR was highlighted and the urgent need for enhanced management of antibiotic usage in veterinary practices were suggested. Baleivanualala et al. studied the extend of environmental contamination in Fijian hospitals by examining carbapenem-resistant *Acinetobacter baumannii* on high-touched surfaces. In their study, phylogenetic links between environmental and clinical isolates were identified. Their work demonstrated the persistence of carbapenem-resistant pathogens on hospital surfaces and the critical role of patients, healthcare workers, and the hospital environment in the transmission of AMR pathogens. Their results pointed out that rigorous infection control measures are needed to prevent hospital-acquired infections.

AMR among vulnerable members of populations is further examined by Khafaja et al. Multidrug-resistant Gram-negative bacterial infections were examined in immunocompromised pediatric patients in Lebanon. Their study identified chemotherapy and thrombocytopenia as significant risk factors. The need for improvement of antimicrobial stewardship programs and regular updates to treatment guidelines for these high-risk groups were emphasized. Liu et al. provided insights into the resistance and virulence profiles of *Streptococcus agalactiae* in dairy farms in northern China. A strong correlation between tetracycline resistance phenotypes and genotypes was demonstrated while the importance of monitoring zoonotic AMR to protect both animal and human health was underlined.

In this Research Topic molecular mechanisms driving AMR were further investigated by several authors. Mendes et al. presented a systematic review linking specific gene mutations in *Neisseria gonorrhoeae* to resistance against key antibiotics. Their work contributed to understanding of resistance mechanisms, particularly the roles of penA and gyrA mutations. Lau et al. highlighted the silent spread of extended-spectrum β-lactamase (ESBL) genotypes in phenotypically susceptible *Escherichia coli* and *Klebsiella pneumoniae* isolates in Malaysia. Their findings raised concerns about the underestimation of AMR in clinical settings. and the inclusion of genotypic testing in routine diagnostics were suggested.

Collectively, the studies in the Research Topic highlight the urgent need for improved infection control protocols and molecular epidemiological analyses to track and mitigate AMR in healthcare settings. These studies emphasize the importance of surveillance, molecular diagnostics, environmental interventions, and community-based strategies to effectively address the AMR issues in developing countries.

